# Pleth variability index versus pulse pressure variation for intraoperative goal-directed fluid therapy in patients undergoing low-to-moderate risk abdominal surgery: a randomized controlled trial

**DOI:** 10.1186/s12871-019-0707-9

**Published:** 2019-03-09

**Authors:** Sean Coeckelenbergh, Amélie Delaporte, Djamal Ghoundiwal, Javad Bidgoli, Jean-François Fils, Denis Schmartz, Philippe Van der Linden

**Affiliations:** 1 Department of Anesthesiology, CHU Brugmann, 4, Place Van Gehuchten, 1020 Bruxelles, Belgium; 2 Université Libre de Bruxelles, Brussels, Belgium; 3 Ars Statistica SPRL, Nivelles, Belgium

**Keywords:** Anesthesia, Anesthesiology, Goal-directed therapy, Colloids, Crystalloids, Fluid responsiveness, Hemodynamics

## Abstract

**Background:**

Goal-directed fluid therapy (GDFT) based on dynamic indicators of fluid responsiveness has been shown to decrease postoperative complications and hospital length of stay (LOS) in patients undergoing major abdominal surgery. The usefulness of this approach still needs to be clarified in low-to-moderate risk abdominal surgery. Both pulse-pressure variation (PPV) and pleth variability index (PVI) can be used to guide GDFT strategies. The objective of this prospective randomized controlled trial was to determine if the use of PVI guided GDFT, when compared to PPV guided GDFT, would lead to similar hospital LOS in patients undergoing low-to-moderate risk surgery. Secondary outcomes included amount of fluid administered and incidence of postoperative complications.

**Methods:**

Patients were randomized into either PVI or PPV guided GDFT groups. Both received a baseline 2 ml kg^− 1^ h^− 1^ Lactated Ringer infusion. Additional fluid boluses consisted of 250 mL of colloid that was infused over a 10 min period if PVI was > 15% or PPV was > 13% for at least five minutes. The primary outcome was to determine if hospital LOS, which was defined as the number of days from surgery up to the day the surgeon authorized hospital discharge, was equivalent between the two groups.

**Results:**

A total of 76 patients were included and they were randomized into two groups of 38 patients. Baseline characteristics were similar in both groups. Both PVI and PPV guided GDFT strategies were equivalent for the primary outcome of LOS (median [interquartile range]) (days) 2.5 [2.0–3.3] vs. 3.0 [2.0–5.0], *p* = 0.230, respectively. Fluids infused, postoperative complications, and all other outcomes were not different between groups.

**Conclusion:**

In patients undergoing low-to-moderate risk abdominal surgery, PVI seems to guide GDFT similarly to PPV in regards to hospital LOS, amount of fluid, and incidence of postoperative complications. However, in low-risk patients undergoing these surgical procedures optimizing stroke volume may have limited impact on outcome.

**Trial registration:**

ClinicalTrials.gov Identifier: NCT02908256, September 2016, retrospectively registered.

**Electronic supplementary material:**

The online version of this article (10.1186/s12871-019-0707-9) contains supplementary material, which is available to authorized users.

## Background

Goal-directed fluid therapy (GDFT), guided by dynamic indicators of fluid responsiveness, (e.g. pulse pressure variation (PPV)) has been shown to decrease postoperative complications and to shorten length of stay (LOS) during major abdominal surgery [1–3]. Dynamic indicators are based on cardiopulmonary interactions during mechanical ventilation and predict fluid responsiveness better than static indicators (e.g. central venous pressure and non-invasive blood pressure) [4–8].

Pulse pressure variation can guide GDFT [9], but it requires the insertion of an arterial catheter and its inherent risks [10]. This is not always desirable, especially for low-to-moderate risk surgeries where blood loss and fluid shifts are minimal. In cases where the risks of an invasive catheter outweigh its potential benefits, pleth variability index (PVI) may be a valuable alternative. PVI is a totally non-invasive monitor which only requires a pulse oximeter [11]. PVI-based GDFT has been shown to reduce postoperative lactate levels and improve postoperative outcome in patients undergoing high-risk abdominal surgery [12–14]. It is safe, easily implemented, and non-invasive. Nevertheless, PVI’s correlation to PPV has been shown to be weak during major abdominal surgery and cardiac surgery, which may be due to intraoperative or chronic changes in arteriolar compliance, sympathetic tone, and heart rate variability [15, 16]. In low-to-moderate risk abdominal surgery, however, such effects on arteriolar and sympathetic tone are probably less frequent and the clinical impact of PVI remains to be determined. PVI may thus be useful in guiding GDFT in this population where these hemodynamic alterations are scarce.

The primary objective of this prospective randomized controlled trial was to demonstrate that the use of PVI guided GDFT and PPV guided GDFT would lead to equivalent hospital LOS in patients undergoing low-to-moderate risk abdominal surgery. Secondary objectives included the amount of fluid administered and the incidence of postoperative complications associated to each GDFT strategy.

## Methods

This single center prospective parallel group randomized controlled trial was performed at the CHU-Brugmann Hospital after ethical committee approval (Brussels, Belgium) (N°CE/201134 and B077201112471) and was registered on clinicaltrials.gov (NCT02908256). Patients gave written informed consent, were adults scheduled for elective low-to-moderate risk abdominal surgery lasting at least one hour, and were allocated into each group after randomization with a ratio of 1:1. Patients were randomized with internet-based software (http://www.randomization.com) and the determined groups were placed in numbered sealed envelopes. AD and DG generated the randomization sequence, enrolled patients, and assigned patients to the interventions. Exclusion criteria consisted of patients younger than 18 years, American Society of Anesthesiologist physical status score greater than III, a body mass index > 35 kg m^− 2^, chronic cardiac arrhythmias, altered myocardial function (left ventricular ejection fraction < 25%), peripheral vascular disease, severe respiratory disease, gelatin allergy, and end stage renal failure (creatinine clearance < 30 ml min^− 1^).

### Anesthesia protocol

Patients fasted at least 6 h for solids and 2 h for clear liquids preoperatively and received oral alprazolam 0.5mg one hour before induction of general anesthesia. Patient monitoring included 5 lead electrocardiogram, non-invasive blood pressure, and peripheral pulse oximetry. The Masimo Radical 7 Set pulse oximeter (Masimo Corp, Irvine, CA, USA) was applied on the index finger, contralateral to the blood pressure cuff, and covered from ambient light for the continuous monitoring of the PVI. A 20-G radial artery catheter was placed ipsilateral to the pulse oximeter after induction of anesthesia. PPV were displayed on the IntelliVue MP5 monitor (Philips Healthcare, Best, Netherlands). Investigators were blinded to the values not pertaining to the allocated GDFT protocol. Entropy sensors (GE Healthcare, Chalfont St Giles, United Kingdom) monitored anesthesia depth and values were kept between 40 and 60 for all patients. Anesthetists induced anesthesia with a propofol bolus of 1–2 mg kg^− 1^ and a sufentanil 0.3 ng ml^− 1^ target controlled infusion Gepts model [17] (Alaris PK syringe pump, CardinalHealth, Rolle, Switzerland). Rocuronium 0.5 mg kg^− 1^ facilitated oral tracheal intubation and muscle paralysis was maintained with additional doses of 0.1 mg kg^− 1^ to maintain a train of four ratio of 0:4 (TOF Watch, Alsevia Pharma, France). Anesthesia was maintained with sevoflurane and sufentanil with a target concentration of 0.2 ng ml^− 1^. Rectal temperature was measured continuously and a forced-air warming blanket was added to maintain normothermia. Tidal volume was maintained at of 8 ml kg^− 1^ of ideal body weight and the respiratory frequency was set to maintain expiratory carbon dioxide between 30 and 35 mmHg. Positive end-expiratory pressure was set to 5 cm H_2_O in all patients. Trendelenburg was set at 10° using a goniometer (iPhone, Apple, Cupertino, CA, USA) that was placed lateral to each patient’s head. Postoperative pain control was achieved with the following analgesics (if not contraindicated): paracetamol, diclofenac, tramadol, and patient controlled intravenous morphine. Use of nitrous oxide, ketamine, or clonidine was prohibited intraoperatively.

### GDFT protocol

**Fig. 1:**
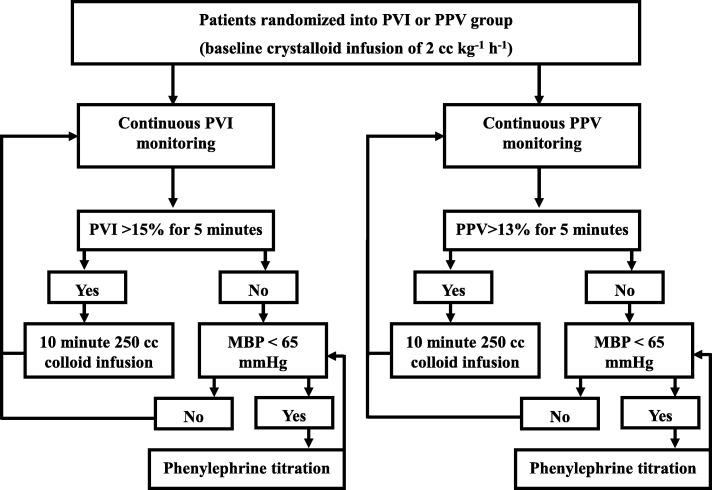
Goal-directed fluid therapy protocols. Patients were randomized into either PVI or PPV guided groups. *PPV pulse pressure variation, PVI Pleth variability index*

Patients were randomized into either PPV GDFT or PVI GDFT groups. Anesthetists were blinded to the monitor not corresponding to their allocated group while patients and postoperative care givers were blinded to the intervention. All patients received a baseline 2 ml kg^− 1^ h^− 1^ Lactated Ringer infusion. The fluid challenge consisted of a 250 ml bolus of colloid (Geloplasma, Fresenius Kabi SA, Belgium) that was infused over a 10 min period. Patients in the PVI GDFT group received a fluid challenge if PVI was higher than 15% for more than 5 min while patients in the PPV GDFT group received a fluid challenge if PPV was higher than 13% for more than 5 min [11, 18, 19]. Boluses were repeated until patients were no longer over the PVI or PPV fluid challenge thresholds. Phenylephrine was titrated if mean arterial blood pressure remained below 65 mmHg despite preload optimization (i.e. PVI or PPV values were below the predetermined fluid responsiveness threshold) (Fig. 1). Additional crystalloids infused for antibiotic and analgesic administration were recorded and added to the total infused volume. Management of acute hemodynamic instability associated with hemorrhage was left at the attending anesthesiologist’s discretion. There was no change in threshold values during pneumoperitoneum and the GDFT protocols were the same regardless of the use of laparoscopy.

### Outcomes variables

The primary outcome was hospital the LOS, which was defined as the number of days from surgery up to the day the surgeon authorized hospital discharge. If patients remained hospitalized longer than the date decided by the surgeon, the surgical discharge date noted in the medical record was still used to calculate hospital LOS. Other outcomes included total infused colloid, total infused crystalloid, estimated blood loss, diuresis, intraoperative use of phenylephrine (i.e., mean arterial blood pressure remained below 65 mmHg despite preload optimization), post-anesthesia care unit (PACU) LOS, number of anti-emetics administered at the PACU, post-operative complications (Additional file 1), time to first ambulation, and postoperative day 1 pain evaluation using visual analogue scale score.

### Statistical analysis

The primary objective of the study was to demonstrate that hospital LOS was equivalent between the two groups. For a difference of one day and equivalence margin of two days a study with a power of 90% and an alpha of 0.01 would require 30 patients per group. Since we estimated a drop-out rate of about 20%, we elected to recruit 38 patients per group. The R package TrialSize was used based on the work of Chow et al. [20]. We planned to determine if groups had comparable variances with Bartlett’s test for homogeneity of variance and if the residuals of the t-test were normally distributed. In the case of non-normal distribution, a non-parametric approach would be used with the R package nparcomp [21] to take into account the non-parametric Behrens-Fisher problem [22]. This package tests whether the observations in one group tend to be different than those of another. If the 95% confidence interval does not contain 0.5 the two groups are significantly different.

The Schapiro-Wilk test determined normality for continuous variables. Normally distributed variables were analyzed with student-t test and non-normal variables were analyzed with Mann-Whitney U test. Categorical variables were analyzed with Chi-square. Statistics were carried out using Minitab statistical software (Paris, France). Group allocation was revealed after data analysis.

## Results

**Fig. 2:**
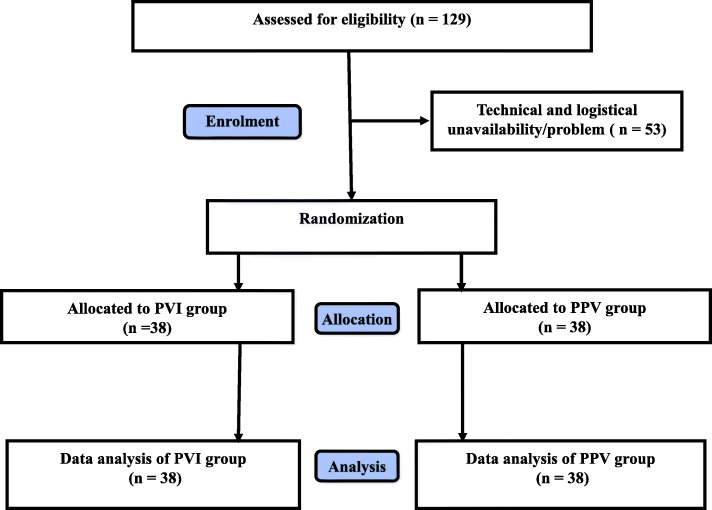
Flow chart of enrolment, allocation, and analysis

**Fig. 3:**
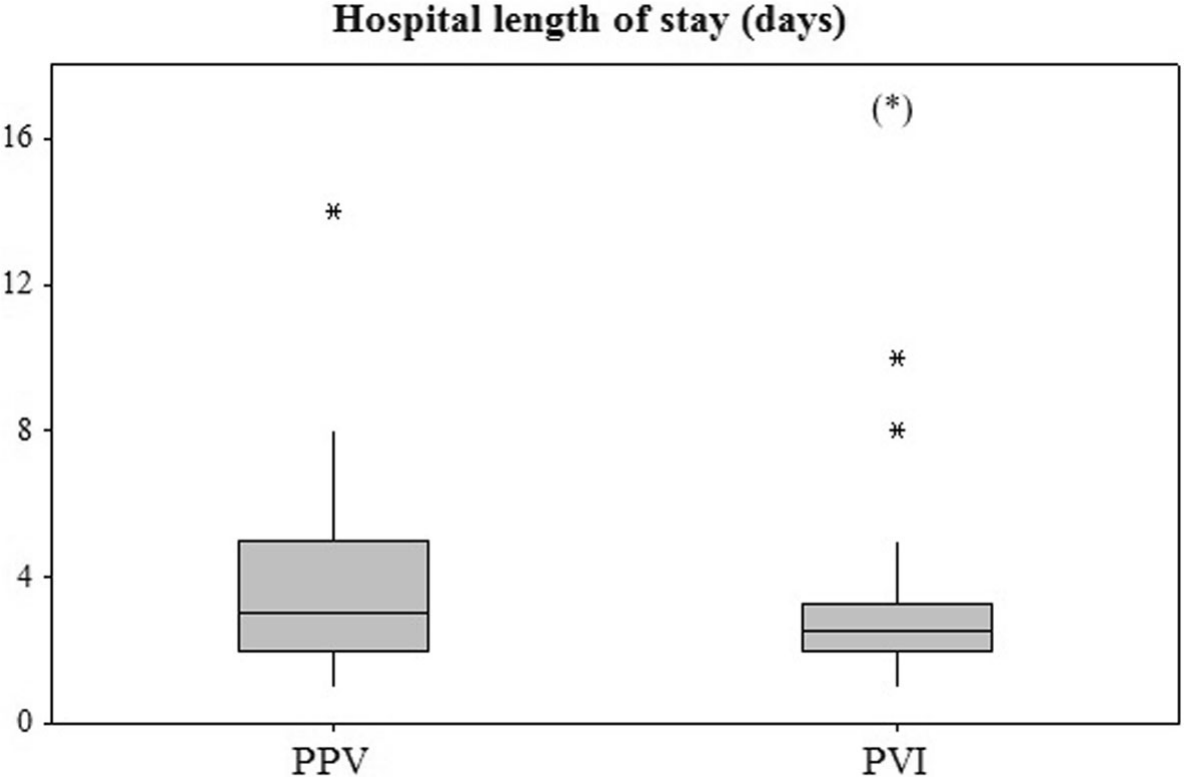
Box-plot comparison of the primary outcome, length of stay (LOS). (*) In order to improve readability, one outlier (LOS = 35 days) is not represented in this figure

A total of 76 patients of the initially 129 screened patients were included from July 2011 to October 2013 (Fig. 2). Baseline characteristics were similar in both groups (Table 1). Anesthesia and surgical times were similar between groups. Patients remained in their preassigned group throughout the study period. Bartlett’s test of homogeneity of variance (Chi^2^ = 19.69, df = 1, *p*-value < 0.001) indicated that variances were not equal between groups and the Shapiro-Wilk normality test indicated that the residuals were not normally distributed for LOS. Therefore, the non-parametric approach was applied, and no difference was observed for the primary outcome of LOS (median [interquartile range]) 2.5 [2.0–3.3] vs. 3.0 [2.0–5.0] days, *p* = 0.230, for PVI and PPV, respectively (Fig. 3). The 95% confidence interval equals [0.291–0.549]. Since it contains the 0.5 value, the two groups are equivalent for LOS.

**Table 1:** Baseline characteristics, expressed as % (number), mean (+/−SD), or median [IRQ]

	PVI (*n* = 38)	PPV (*n* = 38)	*p*-value
Age (year)	45 (+/−13)	50 (+/− 13)	0.124
Female gender	92.1 (35)	94.7 (36)	0.644
Weight (kg)	72 (+/−15)	66 (+/− 15)	0.096
*ASA Score*			0.154
ASA physical status 1	29.0 (11)	44.7 (17)	
ASA physical status 2	71.1 (27)	55.3 (21)	
*Medications %*			
Aspirin	2.6 (1)	10.5 (4)	0.165
Beta-blocker	13.2 (5)	10.5 (4)	0.723
ACEI	7.9 (3)	2.6 (1)	0.304
ARB	5.3 (2)	5.3 (2)	1.00
Calcium channel blocker	5.3 (2)	2.6 (1)	0.556
SSRI	5.3 (2)	5.3 (2)	1.000
Antidepressant other	15.8 (6)	7.9 (3)	0.287
Benzodiazepine	13.2 (5)	15.8 (6)	0.744
Hypertension	31.6 (12)	15.8 (6)	0.105
Diabetes	5.3 (2)	2.6 (1)	0.556
Obstructive pulmonary	5.3 (2)	13.2 (5)	0.234
Previous major surgery	21.1 (8)	23.7 (9)	0.783
*Type of surgery*			0.312
Laparoscopy (%)	65.8 (25)	76.3 (29)	
Laparotomy (%)	34.2 (13)	23.7 (9)	
Surgery Duration (min)	130 (+/− 75)	122 (+/−47)	0.599
Anesthesia Time (min)	180 [136–215]	183 [142–229]	0.670

*ASA* American Society of Anesthesiology, *ACEI* angiotensin converting enzyme inhibitor, *ARB* aldosterone receptor blocker, *SSRI* selective serotonin reuptake inhibitor

Infused crystalloid volume, infused colloid volumes, estimated blood loss, diuresis, time to first ambulation, and pain evaluation at post-operative day 1 were not different between groups (Table 2). The use of phenylephrine to correct hypotension in preload optimized patients was not different between groups. There was no difference in individual postoperative complications or composite postoperative complications. None of the patients required supplementary fluids due to hemorrhage associated hemodynamic instability. No harm was caused by the intervention and no patient died during the study period. No correction was done for missing data.

**Table 2:** Study outcomes, expressed as % (number), mean (+/−SD), or median [IRQ]

	PVI	PPV	*p*-value
*Intraoperative data*			
Total colloid (ml)	500 [0–750]	250 [0–500]	0.275
Total crystalloid (ml)	500 [350–740]	550 [450–663]	0.458
Estimated blood loss (ml)	100 [50–300]	100 [50–163]	0.445
Urine output (ml)	150 [79–250]	150 [100–250]	0.803
Intraoperative phenylephrine	26.3 (10)	21.1 (8)	0.589
*Postoperative data*			
LOS PACU (min)	105 [90–135]	90 [45–135]	0.382
PACU PONV	10.5 (4)	7.9 (3)	0.692
*Number of antiemetics PACU*			0.330
0	89 (34)	92 (35)	
1	5.3 (2)	7.9 (3)	
2	5.3 (2)	0 (0)	
Hypotension PACU	5.3 (2)	7.9 (3)	0.644
VAS Day 1 score	1 [0–2]	2 [0–2]	0.925
Ward PONV	7.9 (3)	7.9 (3)	1.000
Fever	7.9 (3)	2.6 (1)	0.304
Post-operative complications	4 (10.5)	2 (5.3)	0.395
Time to first ambulation	1 [1–2]	2 [1–2]	0.231

*LOS* length of stay, *PONV* postoperative nausea or vomiting, *PACU* post anesthesia care unit, *VAS* visual analogue scale

## Discussion

In patients undergoing low-to-moderate risk abdominal surgery PVI and PPV guided GDFT are considered equivalent for the primary outcome of hospital LOS and no difference was found between the secondary outcomes. Both strategies seem to optimize preload equally and lead to similar outcome. PVI nonetheless has the distinct advantages of being totally non-invasive. Although PPV can be displayed continuously with certain monitors, it is invasive and does not improve outcome when compared to PVI in this population.

Several studies have shown that PVI guided GDFT, when compared to fluid therapy guided by static parameters of fluid responsiveness, can lead to decreased infused fluid volume, decreased time to first stool, and decreased perioperative lactate levels [12, 13, 23, 24]. Trials comparing different GDFT strategies to PVI, such as esophageal Doppler, have however resulted in conflicting results. For example, when compared to esophageal Doppler in patients requiring renal transplantation, PVI was shown to detect fluid responsiveness less consistently [25]. These patients may have pathological endothelial changes affecting arterial compliance that lead to poor capillary distribution. Since PVI is a direct measure of arterial compliance [26], these effects may alter the fluid response threshold. However, when compared to esophageal Doppler GDFT in patients requiring colorectal resection, a population more comparable to ours, fluid administration and outcome was not different [27]. Bahlmann et al. also showed that PVI and stroke volume optimization assessed by esophageal Doppler during open abdominal surgery had similar outcome [28]. Our results parallel the latter studies and indicate that in low-to-moderate risk abdominal surgery, PVI seems to be an adequate guide for GDFT.

It is important to consider, nevertheless, that low-to-moderate risk abdominal surgery may itself explain the lack of difference between groups. Two recent studies on the impact of non-invasively guided GDFT (Clearsight/ccNexfin) in patients undergoing moderate risk abdominal surgery, when compared to restrictive fluid therapy, were unable to demonstrate any difference between groups [29, 30]. Two possible reasons for these findings are that the non-invasive monitor did not adequately optimize cardiac output or that preload optimization has little impact on outcome in low-to-moderate risk abdominal surgery. However, other studies focusing on low-to-moderate risk abdominal surgery reported that perioperative fluid management could still have an impact on postoperative outcome in this particular population indicating that fluid administration should be individualized and goal directed [31].

This randomized controlled trial had several limitations. In addition to the limitations intrinsic to a single center study, we did not measure protocol compliance, which is a key predictor of GDFT impact [3]. However, since both interventions required similar workloads, compliance should have been similar in both groups. In addition, this study was performed according to an intention to treat analysis and our results consequently reflect clinical practice. The vast majority of patients were women undergoing laparoscopic surgery and this should be noted when applying our GDFT strategies to patients. Another limitation inherent to PVI is its dependence on perfusion index, which varies depending on vascular tone [32]. This limitation nonetheless does not seem to have had an impact on our results since no difference was found between PVI and PPV groups in the amount of fluids administered or on outcome in this overwhelmingly healthy population. Another important point to consider is that this lack of difference between groups may be due to the low impact of GDFT on outcome in the studied population which ultimately consisted of low-risk patients undergoing low-to-moderate risk surgery. Nevertheless, physicians who still wish to implement a GDFT strategy for these patients should prefer a non-invasive monitor, such as PVI, to a more invasive monitor.

## Conclusion

In patients undergoing low-to-moderate risk abdominal surgery, PVI seems to guide GDFT similarly to PPV in regards to hospital LOS, amount of fluid, and incidence of postoperative complications. However, in low-risk patients undergoing these surgical procedures optimizing stroke volume may have limited impact on outcome.

## Additional file

Additional file 1: Definitions of postoperative complications. (DOCX 19 kb)

## Abbreviations

ACEI: Angiotensin converting enzyme inhibitor
ARB: Aldosterone receptor blocker
ASA: American Society of Anesthesiology
GDFT: Goal-directed fluid therapy
LOS: Length of stay
PACU: Post anesthesia care unit
PPV: Pulse pressure variation
PVI: Pleth variability index
SSRI: Selective serotonin reuptake inhibitor
VAS: Visual analogue scale
