# Supplementary material for: Pleth variability index versus pulse pressure variation for intraoperative goal-directed fluid therapy in patients undergoing low-to-moderate risk abdominal surgery: a randomized controlled trial
Source: BMC Anesthesiol. 2019 Mar 9;19:34. doi: 10.1186/s12871-019-0707-9 (PMC6408844; doi:10.1186/s12871-019-0707-9)
Supplement: Supplementary file 1 — Definitions of postoperative complications. (DOCX 19 kb) [file 12871_2019_707_MOESM1_ESM.docx]

**Definitions of postoperative complications**

**Major Complications**

***Anastomotic leakage:*** a defect of the intestinal wall at the anastomotic site leading to a communication between the intra- an extra luminal compartments

***Peritonitis***: Infection or inflammation of the peritoneum caused by intestinal perforation, trauma, postoperative infection from drains, or direct spread of infected organ.

***Sepsis:*** defined using criteria prior to the 2016 sepsis definition (i.e., two or more features of the systematic inflammatory response syndrome (SIRS) plus evidence or suspicion of infection).

***Wound dehiscence:*** postoperative rupture of sutures and opening along the site of surgical incision.

***Stoma necrosis:*** ischemia of surgical stoma leading to necrosis

***Bleeding requiring surgery:*** postoperative bleeding requiring reoperation for hemostasis

***Pulmonary embolism:*** mechanical obstruction of pulmonary artery or arteriole confirmed by chest angiographic computerised tomography, ventilation perfusion scintigraphy, and/or autopsy.

***Pulmonary oedema:*** respiratory distress with impaired oxygenation AND radiological evidence of pulmonary oedema requiring diuretic therapy

***Pneumonia:*** The presence of new and/or progressive pulmonary infiltrates on chest radiograph plus two or more of the following:

1. Fever ≥ 38.5°C or postoperative hypothermia <36°C

2. Leucocytosis ≥ 10,000 WBC/mm3 or leucopaenia < 4,000 WBC/mm3

3. Purulent sputum and/or

4. New onset or worsening cough or dyspnoea.

***Acute coronary syndrome:*** increase and gradual decrease in troponin level or a faster increase and decrease of creatine kinase isoenzyme as markers of myocardial necrosis in the company of at least one of the following: ischaemic symptoms, abnormal Q waves on the ECG, ST segment elevation or depression; coronary artery intervention (e.g. coronary angioplasty) or a typical decrease in an elevated troponin level detected at its peak after surgery in a patient without a documented alternative explanation for the troponin elevation.

***Arrhythmia:*** electrocardiograph (ECG) evidence of cardiac rhythm disturbance.

***Acute kidney injury***: according to The Kidney Disease: Improving Global Outcomes (KDIGO) group criteria

***KDIGO 1:*** increase in serum creatinine 0.3 mg/dl or 150% to 200% from baseline (1.5- to 2-fold)

***KDIGO 2:*** increase in serum creatinine to 200% to 300% from baseline (2- to 3-fold)

***KDIGO 3:*** increase in serum creatinine to 300% or more (3-fold) or increase in serum creatinine to more than 4.0 mg/dl or initiation of renal replacement therapy

***Renal replacement therapy:*** determined by the doctor in charge

***90-day readminssion to hospital:*** patient readmitted within 90 days of operation for a complication associated with the operation

***30-day Mortality***: patient death within the 30 days following primary surgery

**Minor Complications**

***Superficial wound infection:***

(1) Infection occurs within 30 days after surgery and

(2) Involves only skin and subcutaneous tissue of the incision and

(3) The patient has at least one of the following:

(a) purulent drainage from the superficial incision

(b) organisms isolated from an aseptically obtained culture of fluid or tissue from the superficial incision

(c) at least one of the following symptoms or signs of infection: pain or tenderness, localised swelling, redness or heat, and superficial incision is deliberately opened by surgeon and is culture positive or not cultured. A culture negative finding does not meet this criterion.

(d) diagnosis of an incisional surgical site infection by a surgeon or attending physician.

***Urinary tract infection:*** positive urine culture of at least 10^5^ colony forming units per millilitre with no more than two species of micro-organisms, and with at least one of the following symptoms or signs: fever (>38°C), urgency, frequency, dysuria, suprapubic tenderness, costovertebral angle pain or tenderness with no other recognised cause.

***Paralytic ileus:*** absence of flatus or stool for the first 48 hours

***Postoperative confusion-delirium:*** acute confusion or personality change with altered vigilance and no pre-existing cause of cognitive impairment.

**Other Definitions:**

***Any blood transfusion***: including red cell, fresh frozen plasma or platelet transfusion, from the star of surgery

***Reoperation:*** re-intervention within the 30 days following primary surgery.

***Postoperative nausea and vomiting:*** nausea or vomiting within 24-48 hours of surgery requiring antiemetic therapy
